# The Incidence of Acute Kidney Injury (AKI) in Critically Ill COVID-19 Patients: A Single-Center Retrospective Cohort Study at a Tertiary Level Hospital in Oman

**DOI:** 10.7759/cureus.40340

**Published:** 2023-06-12

**Authors:** Sulaiman Y Al Abri, Jyoti Burad, Mazin M Al Wahaibi

**Affiliations:** 1 Anesthesia and Intensive Care, Oman Medical Specialty Board, Muscat, OMN; 2 Anesthesia and Intensive Care, Sultan Qaboos University Hospital, Muscat, OMN

**Keywords:** shock, coronavirus disease 2019, renal dialysis, : acute kidney injury, acute respiratory distress syndrome [ards]

## Abstract

Background: Acute kidney injury (AKI) is associated with adverse outcomes in critically ill patients. Coronavirus disease 2019 (COVID-19) affects the renal system frequently and leads to AKI. This study aims to determine the incidence of AKI, risk factors including hyperglycemia, and the requirement for renal dialysis.

Methods: A retrospectively observational study was done at Sultan Qaboos University Hospital between March 2020 and September 2021. A total of 286 adult patients with laboratory-confirmed COVID-19 infection admitted to the intensive care unit (ICU) were included in the study. The patient’s medical records were reviewed. Patients’ baseline demographic characteristics, APACHE score on admission, clinical data including length of stay, oxygenation parameters, ventilator days, shock, AKI (KIDIGO guideline), dialysis, medications, lab on admission as well as during the ICU stay, and the outcome (mortality) were recorded in detail. Follow-up was done till discharge from ICU.

Results: The study population included 68.5% (196/286) males. The median age was 56 years (interquartile range, IQR: 43-66.25). The incidence of AKI was 55.2% (158/286) overall. Out of those who had AKI, 27.2% (43/158), 31.6% (50/158), and 41.1% (65/158) developed AKI stages 1, 2, and 3, respectively. Univariate analysis for the development of AKI showed the following significant variables: age (p=0.005; odds ratio, OR 1.024; 95% confidence interval, CI 1.007-1.041), creatinine level on admission (p=0.012; OR 1.005; 95%CI 1.001-1.008), APACHE score on admission (p<0.001; OR 1.049; 95%CI 1.021-1.077), P/F ratio (p<0.001; OR 0.991; 95%CI 0.987-0.995), nephrotoxic agent (p<0.001; OR 8.556; 95%CI 4.733-15.467), shock (p<0.001; OR 8.690; 95%CI 5.087-14.843), days on the ventilator (p<0.001; OR 1.085; 95%CI 1.043-1.129), and length of stay in ICU (p<0.001; OR 1.082; 95%CI 1.047-1.119). The multivariate analysis confirmed only shock (p=0.004; OR 5.893; 95%CI 1.766-19.664). A total of 41.7% (66/158) of patients received dialysis. Hyperglycemia was not associated with the development of AKI. For patients with AKI, those having high APACHE score (p<0.001), shock (p=0.56; OR 2.326; 95%CI 1.036-5.223), ischemic heart disease (IHD) (p=0.002; OR 9.000; 95%CI 1.923-42.130), and hypertension (p=0.023; OR 2.145; 95%CI 11.125-4.090) were significantly associated with the requirement of dialysis. The mortality was found to be 59.1% (169/286) overall whereas it was 83.5% (132/158) for AKI versus 28.9% (37/158) for non-AKI cases.

Conclusions: A high incidence of AKI for critically ill COVID-19 cases was found in this study. The shock was the only significant predictor for the development of AKI. AKI is associated with high mortality in these patients.

## Introduction

The coronavirus disease 2019 (COVID-19) affected almost all populations across the globe significantly. There has been an increase in the rate of respiratory failure, hospitalizations, and intensive care unit (ICU) admissions due to this disease. Acute kidney injury (AKI) is a common complication of COVID-19, associated with a high mortality rate. It has been reported that AKI in patients with COVID-19 infection ranges from 6.5% to 46% [1], with the highest rates among critically ill patients (23%-81%) [1]. Different definitions of AKI and other populations have resulted in variations in the incidence rate of AKI.

Factors contributing to the development of AKI in patients with COVID-19 include hypoxia, shock, diabetes (established as well as new onset hyperglycemia), direct cytotoxicity of endothelial and tubular-epithelial cells and podocytes, microthrombi and thrombotic microangiopathy, or cardiorenal syndrome due to right ventricular failure [2-4]. In critically ill COVID-19 patients, hyperglycemia was frequently observed in diabetic and non-diabetic patients. Since earlier days, diabetes has been shown to cause microangiopathic changes in the lungs [5]. It might have led to problems in the diffusion of gases across the interstitium of the lung. Also, several studies have shown a higher incidence of AKI in critically ill COVID-19 patients with diabetes [6-7]. This led to the particular interest in investigating the role of diabetes as the etiology of AKI in critically ill COVID-19 patients in this study.

Acute kidney injury can lead to increased ICU length of stay, higher ventilator days, and higher mortality. Critically ill COVID-19 patients with AKI frequently require dialysis. Dialysis procedure carries many risks and complications. For example, hypotension, nausea and vomiting, fever and chills, headache, cramps, chest pain and back pain, hypoglycemia, hematoma, catheter tip migration, pulmonary embolism, arrhythmias, stenosis, and infections [8-9]. Hence it was deemed important to investigate AKI in critically ill COVID-19 patients and find out the possible etiological factors associated with AKI and the need for dialysis. Limited studies in the Middle East investigated the incidence of AKI in critically ill COVID-19 patients. This study aimed to find the incidence of AKI, the association of hyperglycemia and other risk factors associated, with AKI, and study the predictors and outcomes of the dialysis requirement for critically ill COVID-19 patients with AKI.

## Materials and methods

Study design and setting

This is a single-center, retrospective cohort study involving adult ICU patients with exposure to COVID-19 infection admitted to the university hospital in Oman between March 2020 and September 2021. The patients were followed till discharge from the ICU for up to 44 days. Data were collected for these patients retrospectively using the hospital information system "Trackcare."

Ethical aspects

This retrospective cohort study was approved by the Medical Research Ethics Committee (MREC), College of Medicine and health sciences, Sultan Qaboos University (MREC#2602). Informed consent was not required due to the retrospective nature of the study. The patient's privacy was always respected by coding the patient's identity. Declaration of Helsinki (1975) and instructions of the institutional medical research ethics committee were strictly followed to veil the patient's identity. The patient data were kept securely with password protection with the investigators. This study was registered with Clinicaltrials.gov (NCT05467956).

Participants 

All patients admitted to COVID-ICU during the study period were scanned. The inclusion criteria were all adult patients admitted to ICU with laboratory-confirmed severe acute respiratory syndrome coronavirus 2 (SARS-CoV-2) infection, documented by real-time reverse transcriptase-polymerase chain reaction (RT-PCR) on nasopharyngeal swabs or lower respiratory tract aspirates. At the same time, exclusion criteria were: patients with a pre-existing renal disease with an estimated glomerular filtration rate (eGFR) < 30 or on dialysis before admission to ICU and insufficient clinical documentation available for review, including loss of follow-up due to transfer to another hospital.

Variables

The primary outcome was the incidence of AKI in critically ill COVID-19 cases. The secondary outcomes were to study the association of glycemic status with AKI in COVID-19 ICU patients and the predictors and outcomes of the proportion of patients requiring dialysis. All the patients had exposure to COVID-19 infection. The predictors of the outcomes studied were glycemic status at admission and shock, whereas hypertension, diabetes mellitus, ischemic heart disease (IHD), age, gender, disease severity through inflammatory markers, and APACHE score were considered confounders. Hypoxia and nephrotoxic medications were considered effect modifiers.

Diagnostic criteria for AKI

Kidney Disease Improving Global Outcomes (KDIGO) criteria were used for AKI diagnosis and stratification [1]: Stage 1, increment of serum creatinine from 0.3 mg/dL in 48 h or increase from 1.5 to 1.9 times the baseline serum creatinine value within 7 days. Stage 2 is a 2- to 2.9-fold increase in serum creatinine within 7 days or urine output below 0.5 mL/kg/h for more than 12 h. Stage 3 is a three-fold increase in serum creatinine in 7 days, creatinine higher than 4 mg/dL, renal dialysis initiation through hemodialysis, urine output below 0.3 mL/kg/h for 24 h or more, or anuria for 12 h or more. The creatinine level was measured as a baseline on admission to the ICU. KIDIGO criteria were used to compare daily creatinine levels from the baseline value, thereby diagnosing and categorizing the AKI.

Bias 

To avoid bias, variation in creatinine level from baseline was considered instead of urine output as only 24-h output charting was available on the system, whereas creatinine was measured at least once daily for all patients. KIDIGO criteria were chosen for accurate diagnosis of AKI as it is highly sensitive and specific for the diagnosis of AKI [10]. Patients transferred to other hospitals while in ICU were lost to follow-up and hence excluded.

Study size

The sample size was estimated based on the expected incidence proportion of AKI among COVID-19 patients. A recently published systematic review has reported a pooled incidence proportion of 19.45% (95% CI; 14.63-24.77) [11]. Therefore, the estimated sample size is based on an expected incidence proportion of 20%, a margin error of 5%, and a confidence level of 95%. The estimated sample size was 250. We could manage to include 286 patients during the study period.

Statistical analysis

Mean, median, and standard deviation were used to describe continuous variables, whereas frequency and percentage were used to describe categorical variables. Unpaired t-tests were used to compare the means of the two groups. A chi-square test (Fisher's exact/Likelihood ratio) was used to evaluate the association between two categorical variables. A multivariate binary logistic regression analysis was used to identify the independent predictors of AKI. A univariate analysis was used to find the significant factors associated with the dialysis requirement. A p-value of 0.05 or less was considered statistically significant. The IBM SPSS Statistics version 28.0 program (IBM Corp., Armonk, NY) was used to conduct the analysis.

## Results

Participants

**Figure 1:**
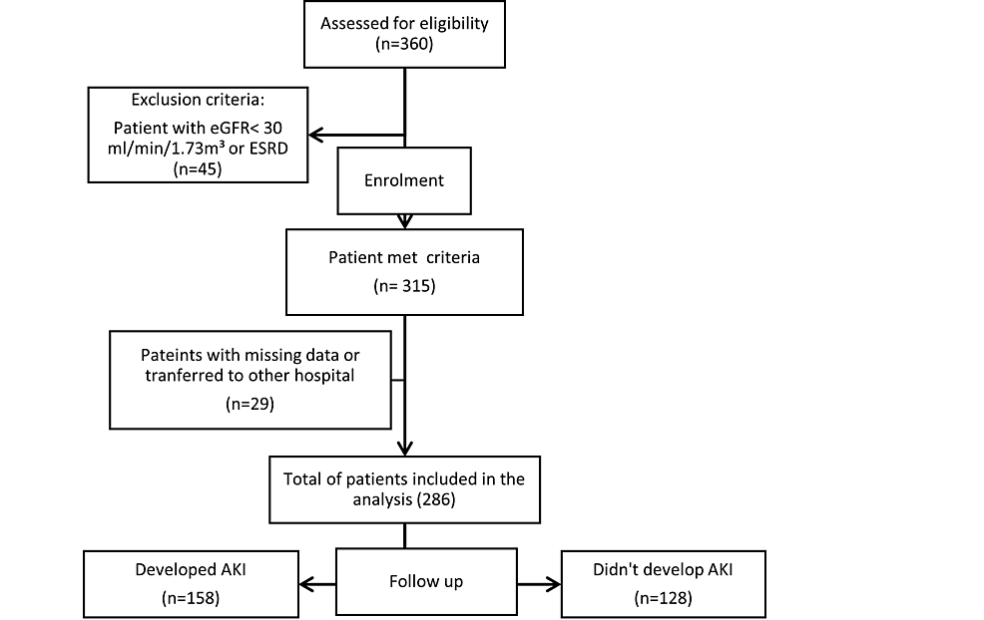
Patient flow. eGFR, estimated glomerular filtration rate; ESRD, end stage renal disease; AKI, acute kidney injury

From March 2020 to September 2021, the records of all 360 adult patients with COVID-19 infection admitted to the ICU were scanned. Of these, 45 patients were excluded from the study due to exclusion criteria (eGFR < 30 mL/min/1.73 m³ or end stage renal disease, ESRD). Of the remaining 310 patients, 29 were missing data or transferred to another hospital, which was difficult to follow up with, and hence were excluded from the study. Finally, 286 patients were studied and included in the statistical analysis (Figure 1).

The patients were followed up highest up to 44 days (the full ICU length of stay).

Descriptive data

The median age among all patients was 56 years (IQR: 24-92 years), and (196/286) 73% were males. Hypertension (HTN) and diabetes mellitus (DM) were the most prevalent diseases found in this cohort, 42% and 38.8%, respectively. Some 69.2% (198/286) of patients received nephrotoxic agents in the form of medications (including antibiotics like vancomycin, colistin, aminoglycosides, antiviral drugs like acyclovir, and anti-fungal like amphotericin) or IV contrast for radiological scans. Only (17/286) 5.9% of patients had preexisting lung diseases. The median P/F ratio was 95.45 (IQR: 51-117.25). Around half of the patients got hyperglycemia (above 10 mmol/L) at least once during the ICU stay. The median stay time in ICU was 9 days (IQR: 1-44 days). Some 55.2% (158/286) of patients developed AKI, of which 41.8% received dialysis. Among all the patients, 23.1% required dialysis, and 54.5% (156/286) of patients developed shock. The overall mortality was 59.1% (169/286) (Table 1).

**Table 1:** General characteristics of the critically ill COVID-19 patients. IQR, interquartile range; SD, standard deviation; n, number; ICU, intensive care unit; APACHE score, the Acute Physiology and Chronic Health Evaluation score; HTN, hypertension; DM, diabetes mellitus; IHD, ischemic heart disease; RBS, random blood sugar; P/F ratio, the arterial partial pressure of oxygen (PaO2) / the inspired oxygen concentration (FiO2); CRP, C-reactive protein; MV, mechanical ventilator

Variables		All patients (n= 286)
Age, median (IQR)	56 (43-66.25)	
Male, (n)%	(196) 68.5%	
APACHE score, median (IQR)	95.45 (51-117.25)	
HTN, (n)%	(120) 42%	
DM, (n)%	(111) 38.3%	
IHD, (n)%	(28) 9.8%	
Lung disease, (n)%	(17) 5.9%	
Shock, (n)%	(156) 54.5%	
Nephrotoxic agents, (n)%	(198) 69.2%	
Creatinine level on ICU admission, Mean (SD)	108.38 ± 132.88	
RBS on ICU admission, mean (SD)	10.626 ± 4.00	
P/F ratio, mean (SD)	95.45 ± 70.72	
CRP, mean (SD)	147.27 ± 114.40	
D-dimer, mean (SD)	9.01 ± 15.52	
Hyperglycemia, (n)%	(141) 49.3%	
Hemodialysis, (n)%	(66) 23.1%	
Days in ICU, median (IQR)	9 (5-17)	
Days on MV, median (IQR)	8 (5-15)	
Death, (n)%	(169) 59.1%	

The mortality was 83.5% for AKI vs. 28.9% for non-AKI cases.

Other analysis

Characteristics of AKI patients (n=158): The number of males was (109/158) 69%. The mean age was 52.2 ± 14.51 years. The mean APACHE score was 20.65 ± 9.29. Total 88% (139/158); p<0.001 patients who received nephrotoxic agents developed AKI. The mean creatinine level on the day of ICU admission was (127.13 ± 172.30). The mean of the P/F ratio was 78.51 ± 55.01. A total (80/158) of 50.6% of patients were found to have hyperglycemia. Some 41.8% (66/158) of patients required dialysis. The average length of stay in the ICU was 13.57 ± 8.57 days, and the average ventilation days were 12.03 ± 7.44 days. The mortality was found to be 83.5% (132/158) (Table 2).

**Table 2:** Comparison of baseline characteristics, laboratory data, and treatments between AKI and no-AKI groups. SD, standard deviation; n, number; ICU, intensive care unit; APACHE score, the Acute Physiology and Chronic Health Evaluation score; HTN, hypertension; DM, diabetes mellitus; IHD, ischemic heart disease; RBS, random blood sugar; P/F ratio, the arterial partial pressure of oxygen (PaO2) / the inspired oxygen concentration (FiO2); CRP, C-reactive protein; MV, mechanical ventilator; AKI, acute kidney injury

Variables		p-value		AKI (n = 158)	No AKI (n = 128)
Age, mean (SD)		0.005		52.22 ± 14.51	57.16 ± 14.55
Male, n (%)		0.898		109 (69%)	87 (68 %)
APACHE score, mean (SD)		<0.001		20.65 ± 9.29	16.67 ± 9.09
HTN, n (%)		0.548		69 (43.7%)	51 (40%)
DM, n (%)		0.715		63 (40%)	48 (37.5%)
IHD, n (%)		0.424		13 (8.2%)	15 (11.7%)
Lung disease, n (%)		0.462		11 (6.7%)	6 (4.7%)
Shock, n (%)		<0.001		121 (76.6%)	35 (27.3%)
Nephrotoxic agents, n (%)		<0.001		139 (88%)	59 (46%)
Creatinine level on ICU admission, mean (SD)		0.004		127.13 ± 172.30	85.24 ± 44.13
RBS on ICU admission		0.636		10.75 ± 3.65	10.45 ± 4.42
P/F ratio, mean (SD)		<0.001		78.51 ± 55.01	116.37 ± 81.76
CRP, mean (SD)		0.085		157.36 ± 123.88	134.30±99.94
D-dimer, mean (SD)		0.072		10.45 ± 17.57	7.09 ± 12.10
Hyperglycemia, n (%)		0.363		80 (50.6%)	61 (47.7%)
Hemodialysis, n (%)		<0.001		66 (41.8%)	0
Days in ICU		<0.001		13.57 ± 8.57	8.65 ± 7.65
Days on MV		<0.001		12.03 ± 7.44	8.12 ± 6.81
Death, n (%)		<0.001		132 (83.5%)	37 (28.9%)

Missing data

Some 14% (40/286) for Ferritin, 46.7% (133/286) for body mass index (BMI), 1.74% (5/286) for C-reactive protein (CRP), 62.2% (178/286) for IL6, and 10.8% (31/286) for D-dimer, 8% (23/286) for days on the ventilator, and 0.35% (1/286) for the length of stay in ICU. The variables with missing data >10% were excluded from the analysis (multivariate).

Outcome data

Primary Outcome

**Figure 2:**
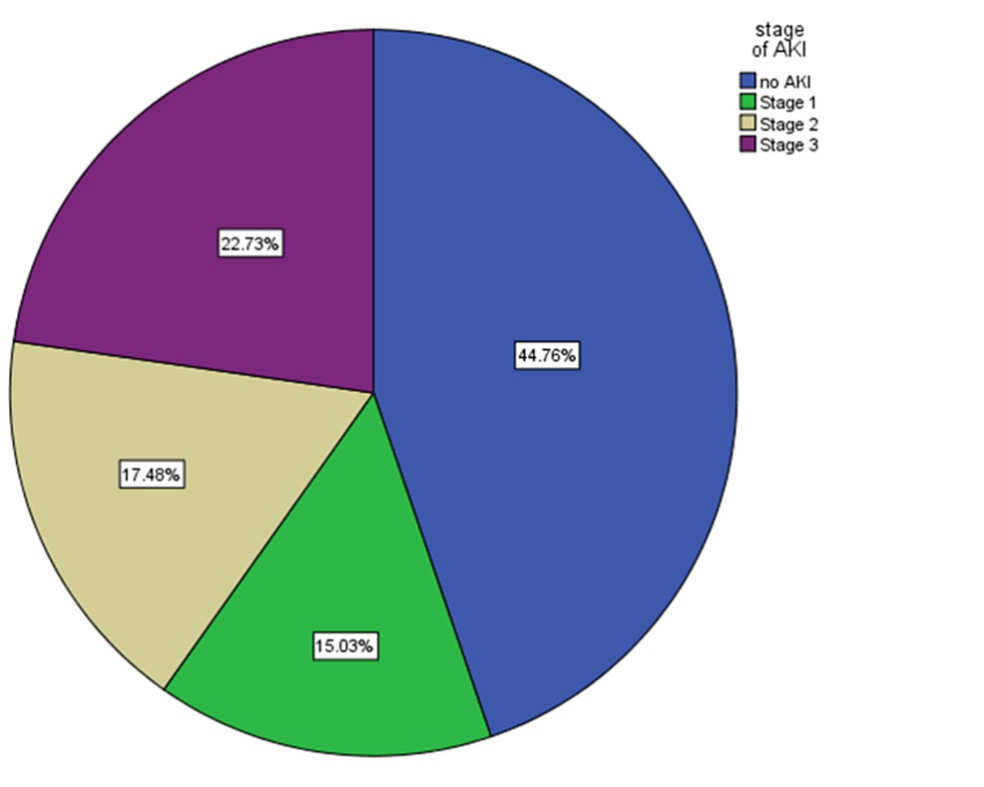
Incidence of different stages of AKI. AKI, acute kidney injury

A total of 55.2% (158/286) of the patients developed AKI. Out of all AKI patients, 27.2% (43/158) had stage 1, 31.6% (50/158) had stage 2, and 41.1% (65/158) had stage 3 AKI (Figure 2).

**Figure 3:**
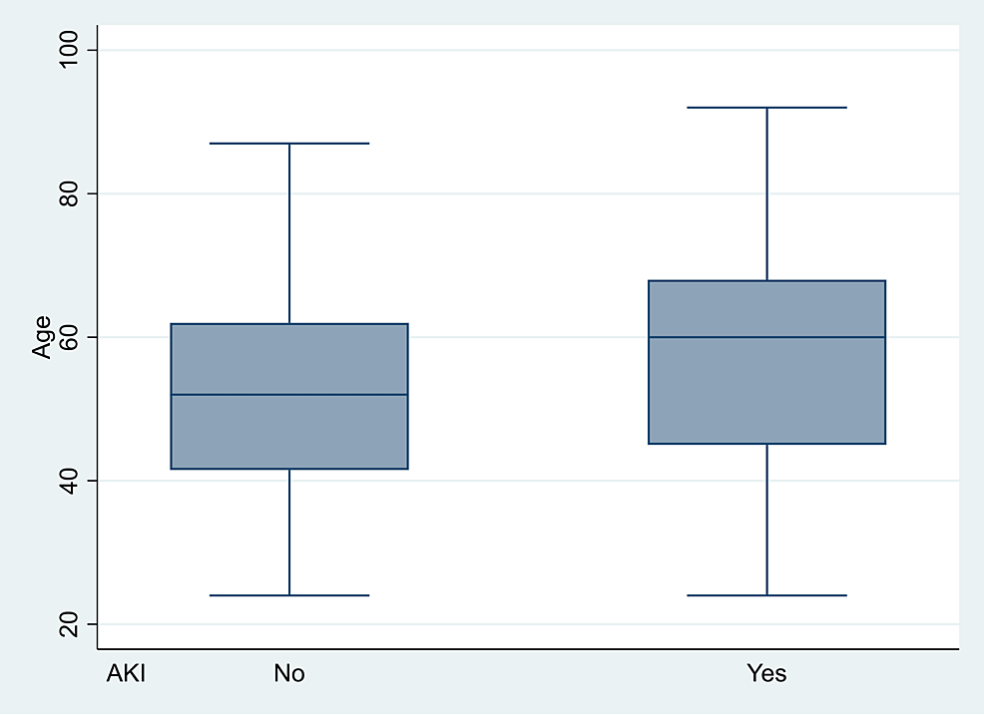
Association between AKI and age. AKI, acute kidney injury

**Figure 4:**
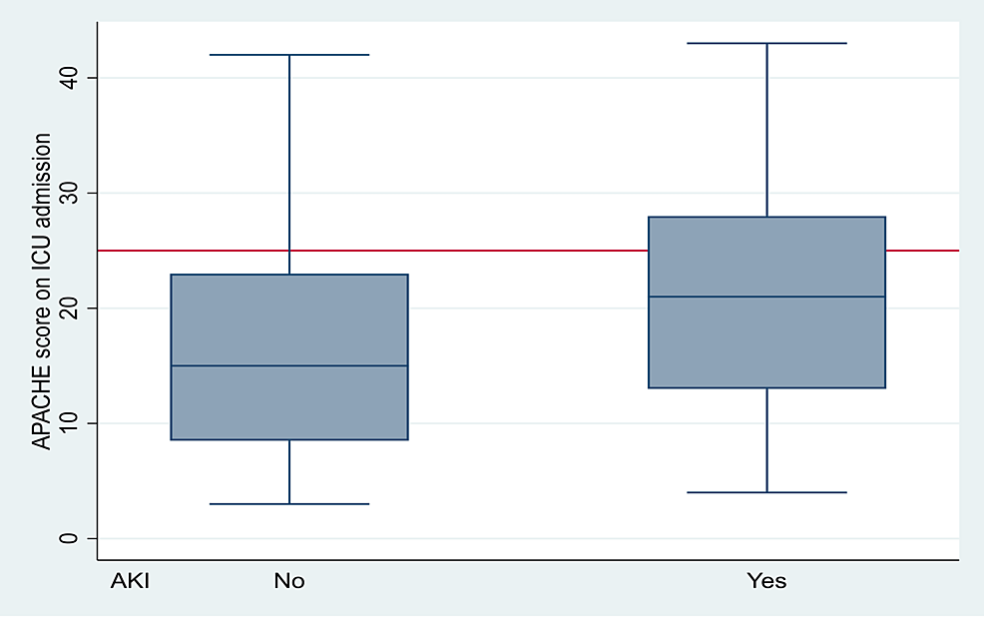
Association between AKI and APACHE 2 score. AKI, acute kidney injury; APACHE, the Acute Physiologic Assessment and Chronic Health Evaluation

**Figure 5:**
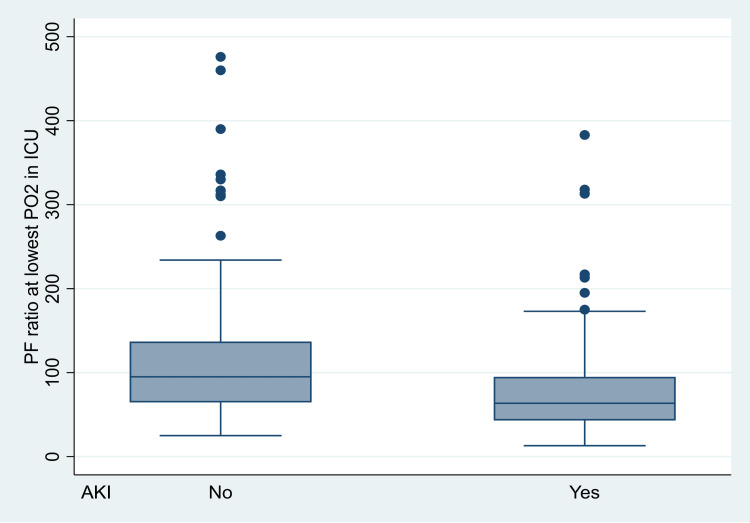
The association between AKI and P/F ratio at the lowest PO2. AKI, acute kidney injury; P/F, partial pressure of oxygen/fraction of inspired oxygen; PO2, partial pressure of oxygen

**Figure 6:**
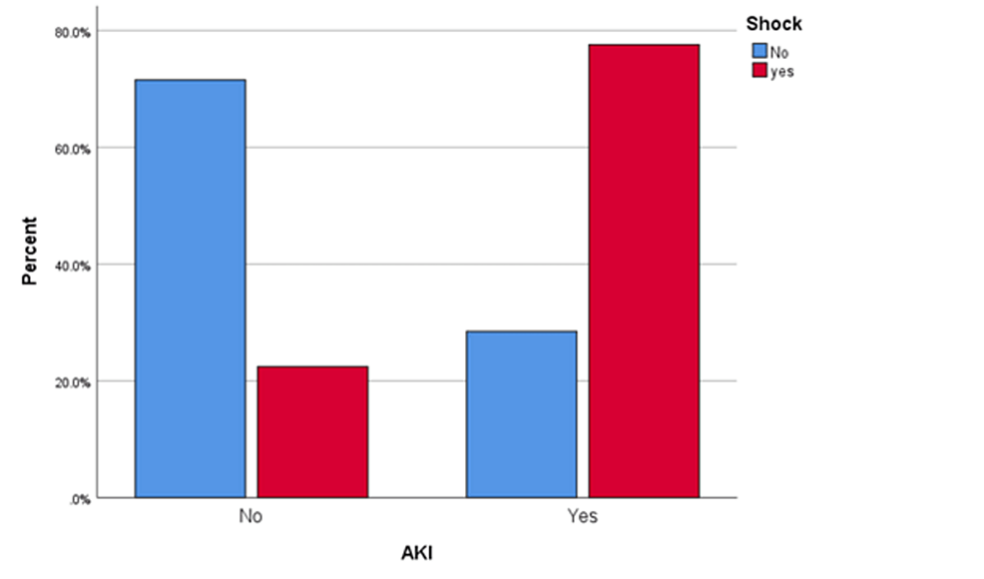
Association between AKI and shock. AKI, acute kidney injury

Univariate analysis of the predictors and confounders for the development of AKI showed the following significant variables: age (p = 0.005, OR 1.024, 95%CI 1.007-1.041) (Figure 3),

creatinine level on admission (p=0.012, OR 1.005, 95%CI 1.001-1.008), APACHE score on admission (p<0.001, OR 1.049, 95%CI 1.021-1.077) (Figure 4),

P/F ratio (p<0.001, OR 0.991, 95%CI 0.987-0.995) (Figure 5),

nephrotoxic agent (p<0.001, OR 8.556, 95%CI 4.733-15.467), shock (p<0.001, OR 8.690, 95%CI 5.087-14.843) (Figure 6),

Days on the ventilator (p<0.001, OR 1.085, 95%CI 1.043-1.129), and length of stay in ICU (p<0.001, OR 1.082, 95%CI 1.047-1.119). Hence, these variables were included in multivariate analysis, which confirmed only shock (p=0.004, OR 5.893, 95%CI 1.766-19.664) as the significant risk factor for the development of AKI in critically ill COVID-19 patients (Table 3).

**Table 3:** Univariate and multivariate logistic regression analysis of risk factors associated with the development of AKI among critically ill COVID-19 patients. 95% CI, 95% confidence interval; OR, odds ratio; ICU, intensive care unit; APACHE score, the Acute Physiology and Chronic Health Evaluation score; HTN, hypertension; DM, diabetes mellitus; IHD, ischemic heart disease; RBS, random blood sugar; P/F ratio, the arterial partial pressure of oxygen (PaO2) / the inspired oxygen concentration (FiO2); CRP, C-reactive protein; MV, mechanical ventilator

Variable	Unadjusted OR	95% CI	p-value	Adjusted OR	95% CI	p-value
Age	1.024	1.007-1.041	0.005	1.013	0.969-1.059	0.574
DM	1.105	0.685-1.784	0.682	0.831	0.194-3.563	0.804
HTN	1.171	0.729-1.879	0.514	1.088	0.238-4.978	0.914
IHD	0.675	0.309-1.477	0.326	0.328	0.047-2.294	0.261
Lung disease	1.522	0.547-4.233	0.421	0.647	0.092-4.548	0.662
Creatinine on ICU admission	1.005	1.001-1.008	0.012	1.002	0.998-1.006	0.366
RBS on ICU admission	1.017	0.959-1.079	0.569	1.058	0.839-1.333	0.635
APACHE score on ICU admission	1.049	1.021-1.077	<0.001	1.002	0.937-1.071	0.959
CRP on ICU admission	1.002	1.000-1.004	0.095	1.000	0.995-1.004	0.905
D-dimer on ICU admission	1.015	0.997-1.034	0.093	1.016	0.975-1.060	0.441
PF ratio at lowest PO2 in ICU	0.991	0.987-0.995	<0.001	0.996	0.985-1.008	0.535
Hyperglycemia	1.127	0.707-1.796	0.617	0.374	0.061-2.299	0.288
Nephrotoxic agents	8.556	4.733-15.467	<0.001	3.234	0.743-14.068	0.118
Shock during ICU stay	8.690	5.087-14.843	<0.001	5.893	1.766-19.664	0.004
Days on MV	1.085	1.043-1.129	<0.001	1.053	0.849-1.305	0.640
Length of stay in ICU	1.082	1.047-1.119	<0.001	0.959	0.786-1.170	0.678

Secondary outcome

Hyperglycemia

**Figure 7:**
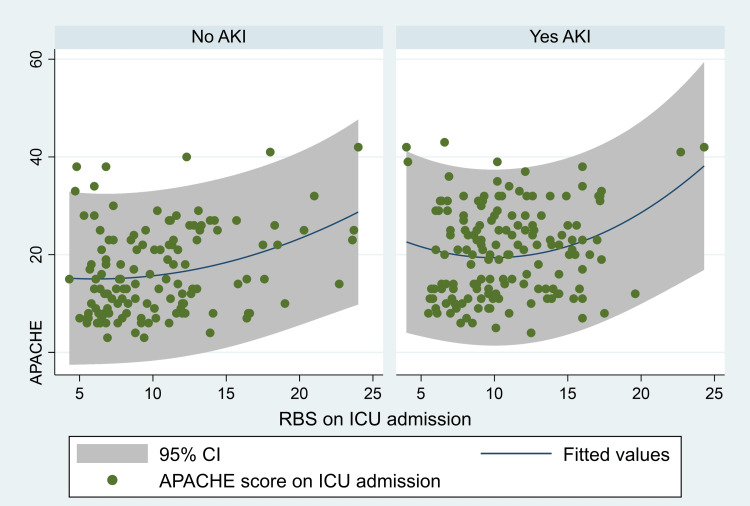
The association between AKI, RBS, and APACHE score. AKI, acute kidney injury; RBS, random blood sugar; APACHE, the Acute Physiologic Assessment and Chronic Health Evaluation

No association of hyperglycemia was found with the development of AKI in this study (p=0.17, OR 1.127, 95%CI 0.707-1.796) (Figure 7).

Requirement of Dialysis

For the patients who developed AKI, those who had a high APACHE score, shock (p=0.56; OR 2.326; 95%CI 1.036-5.223), IHD (p=0.002; OR 9.000; 95%CI 1.923-42.130), and hypertension (p=0.023; OR 2.145; 95%CI 11.125-4.090) had a significantly higher requirement of dialysis.

## Discussion

The incidence of AKI was 55.2% in this study of critically ill COVID-19 patients. The only independent factor linked to the development of AKI was shock. Hyperglycemia was not associated with the development of AKI. This study also showed 41.8% of AKI patients required dialysis.

Incidence of AKI

In critically ill patients admitted to the ICU, the reported incidence of AKI using the KDIGO criteria ranged from 50% to 80% [3-4, 12-13]. In a retrospective analysis of 100 COVID-19 ICU patients, Ghonemy et al. observed a 37% incidence of severe AKI (stages 2 and 3) [9]. Whereas other investigators observed the incidence of severe AKI from 40.6% to 57.4% [3-4, 13-14]. This variation in the incidence could be due to differences in classification scores for AKI and severity of illness as ICU admission criteria.

The severity of AKI and dialysis requirement: This study found a 40.2% incidence of severe AKI stages 2 and 3 (Figure 2). The severity of the COVID-19 patients and the various approaches/definitions used to calculate missing baseline creatinine can cause discrepancies in the reported incidence rates of severe AKI up to 15% [15]. In the present study, the incidence rate of patients requiring dialysis was higher (41.8%) than that reported in the studies of Joseph et al., Doher et al., and Fominskiy et al. (13%, 16.9%, and 17.7%, respectively) and many others [3-4, 12-14, 16]. This discrepancy in the results can be explained by the use of KDIGO guidelines for strict staging, and the fact that the patients admitted to ICU in this study were sicker and almost all required mechanical ventilation in contrast to other studies.

Risk factors for AKI

Different studies show different risk factors for developing AKI [3-4, 12-13]. Hassanein et al. found that AKI was independently associated with older age, vasopressor medications and the need for invasive mechanical ventilation, and the presence of comorbidities (cardiovascular disease, HTN, and DM) in hospitalized COVID-19 patients (n = 5449) in New York [4]. Pan et al., in a retrospective study of COVID-19 patients, found that chronic renal disease, male gender, and admission potassium levels were independent predictors of severe AKI (stage 3) [3]. In another study, chronic renal disease and a modified SOFA score at admission were independently linked with the development of AKI in COVID-19 ICU patients [12]. Another study observed an independent association of diuretics, invasive mechanical ventilation, and creatinine level at admission with AKI in COVID-19 ICU patients [13]. In this study, the shock was associated with AKI (Figure 6). One study shows a significant incidence of AKI following cardiogenic shock, ranging from 20% to 35% [17]. In 5079 patients in Denmark with cardiogenic shock, 13% developed AKI requiring dialysis [18]. The in-hospital mortality was 62% for those who needed dialysis and 36% for those who did not; this result was further supported by a 5-year follow-up analysis, which showed a mortality of 43% for the first group and 29% for the second. As a conformation of cardiogenic shock, AKI was frequently found to be an independent predictor of mortality [19-20]. No relationship was found between hyperglycemia in critically ill COVID-19 patients and the development of AKI (Figure 7). In the NICE-SUGAR trial, which compared two groups of ICU patients, the first group was kept in strict glucose control (81-108 mg/dL), and the second group's blood sugar was kept between 108 and below 180 mg/dL). Around three thousand patients in each group were assigned. The result showed no difference in AKI between the two groups; however, survival was lower in the first group (n = 829-27.5% versus 751-24.9% with p = 0.02) [8]. 

Markers of severity and AKI

A hyperinflammatory condition induced by SARS-CoV-2 infection and high cytokine levels (cytokine storm) have been seen in COVID-19 [21]. It was discovered that a higher rate of death and the development of severe COVID-19 infection were correlated with high levels of IL-6 [22]. It is suspected that IL-6, by causing endothelial and tubular dysfunction, contributes to AKI in COVID-19 patients. Indeed, several models of AKI, such as ischemia and sepsis-induced AKI, have been caused by the harmful effect of IL-6 [22]. IL-6, CRP, and ferritin were not independently linked to AKI in this research (Table 3). The results of this study are consistent with those of Joseph et al., who observed no relationship between the development of AKI and ferritin or IL-6 [12]. The invoked "cytokine storm" induced by COVID-19 infection has been recently challenged. It has been found that even while COVID-19 patients' IL-6 levels were high, they had lower than the average incidence of acute respiratory distress syndrome [23]. However, focused studies are required to confirm/counter this finding.

According to several reports, patients with COVID-19 have a significant incidence of acute thrombotic events and a hypercoagulable condition [24-25]. The development of AKI may be influenced by coagulation dysregulation, as evidenced by thrombi in glomerular loops [25]. But the lack of a correlation between D-dimer and AKI in this research and other publications [12] refutes the notion that hypercoagulability plays a part in COVID-19-induced AKI. Up to this point, there has not been any proof that sepsis-associated AKI and COVID-19 AKI have different pathophysiological pathways [23]. Acute tubular necrosis was the main pathologic finding in kidney biopsies and autopsies of COVID-19 cases [26-27]. The course of treatment should continue to be supportive, with careful fluid and hemodynamic control, avoidance of potentially nephrotoxic substances, and using dialysis individually.

Mortality

Pan et al. observed that AKI was independently associated with death in COVID-19 ICU patients [3]. This study observed a high mortality rate in the AKI group (78.1% death; p < 0.001). Another study found that AKI was positively associated with hospital mortality in 201 critically sick COVID-19 patients [13]. On the other hand, among 330 severely ill COVID-19 patients admitted to the ICU, Vittinghoff et al. did not find a significant correlation between dialysis and death (adjusted OR = 2.3; 95% CI: 0.98-5.5) [28].

Advantages

This is the first study on AKI in critically ill COVID-19 patients in the Middle East population. A proper univariate and multivariate analysis has been done, including many common risk factors.

Limitations

First, it was retrospective research, which means there was a chance of selection bias. Despite this, we identified possible risk factors for AKI and showed that it was an independent predictor of hospital mortality after carefully adjusting for various confounders. Second, it is a single-center study conducted in Oman, and hence the findings might differ in the population of other countries. Third, this study did not include the relationship between the overtime change of renal function during the ICU stay and the development of AKI, the requirement for dialysis, and mortality.

Generalizability

This study used a well-validated AKI risk scoring, applicable to all parts of the world; hence, it can be replicated. However, the results in different geographical areas may vary due to population differences.

## Conclusions

In critically ill patients with COVID-19, AKI was found to be significantly common and was found to be associated with shock but not hyperglycemia. A high proportion of patients with AKI required dialysis in this study. Those with HTN, DM, high APACHE scores, and shock seemed to require dialysis more frequently. As dialysis was not studied as the primary outcome in our research, focused studies are needed to confirm these results.
